# The cost-effectiveness and cost-utility of at-home infrared temperature monitoring in reducing the incidence of foot ulcer recurrence in patients with diabetes (DIATEMP): study protocol for a randomized controlled trial

**DOI:** 10.1186/s13063-018-2890-2

**Published:** 2018-09-24

**Authors:** Wouter B. aan de Stegge, Nora Mejaiti, Jaap J. van Netten, Marcel G. W. Dijkgraaf, Jeff G. van Baal, Tessa E. Busch-Westbroek, Sicco A. Bus

**Affiliations:** 1 Department of Rehabilitation, Amsterdam UMC, location Academic Medical Center, University of Amsterdam, Amsterdam Movement Sciences, Meibergdreef 9, 1105 AZ Amsterdam, The Netherlands; 2 Department of Surgery, Hospital Group Twente, PO Box 7600, 7600 SZ Almelo, The Netherlands; 3 Clinical Research Unit, Amsterdam UMC, location Academic Medical Center, University of Amsterdam, Meibergdreef 9, 1105 AZ Amsterdam, The Netherlands; 4 School of Medicine, Cardiff University, UHW Main Building, Health Park, Cardiff, CF14 4XN Wales, UK

**Keywords:** Diabetes mellitus, Diabetic foot ulcer, Ulcer recurrence, Diabetic foot, Prevention, Home-monitoring, Foot temperature, Cost-effectiveness, Cost-utility

## Abstract

**Background:**

Home monitoring of foot temperatures in high-risk diabetes patients proves to be a promising approach for early recognition and treatment of pre-signs of ulceration, and thereby ulcer prevention. Despite previous studies demonstrating its efficacy, it is currently not widely applied in (Dutch) health care.

**Methods:**

In a multicenter, outcome-assessor-blinded, randomized controlled trial, 304 patients with diabetes mellitus types I or II, loss of protective sensation based on peripheral neuropathy, and a history of foot ulceration in the preceding 4 years or a diagnosis of Charcot neuro-osteoarthropathy will be included. Enhanced therapy will consist of usual care and additional at-home daily measurement of foot temperatures at six to eight predefined locations on the foot. If a contralateral foot temperature difference of > 2.2 °C is found on two consecutive days, the participant is instructed to contact their podiatrist for further foot diagnosis or treatment, and to reduce ambulatory activity by 50% until temperatures are normalized. Enhanced therapy will be compared to usual care. The primary outcomes are the cost (savings) per patient without a foot ulcer (i.e., cost-effectiveness) and per quality-adjusted life year gained (i.e., cost-utility). The primary clinical outcome in the study is the proportion of patients with foot ulcer recurrence on the plantar foot, apical surfaces of the toes, the interdigital spaces or medial and lateral forefoot surfaces during 18-month follow-up.

**Discussion:**

Confirmation of the efficacy of at-home foot temperature monitoring in ulcer prevention, together with assessing its usability, cost-effectiveness and cost-utility, could lead to implementation in Dutch health care, and in many settings across the world.

**Trial registration:**

Netherlands Trial Registration: NTR5403. Registered on 8 September 2015.

**Electronic supplementary material:**

The online version of this article (10.1186/s13063-018-2890-2) contains supplementary material, which is available to authorized users.

## Background

Despite many recent advances in medical therapies, the prevalence of diabetes mellitus and diabetes-related complications continues to increase. With a life-time prevalence of 19–34% [1], foot ulceration is one of the most common complications in people with diabetes. This frequently leads to hospitalization and lower-extremity amputation [2]. With an annual incidence rate of 2.2% and one million people with diabetes, approximately 22,000 ulcers develop in the Netherlands each year [3, 4]. Foot ulcers frequently become infected, cause great morbidity and have a negative impact on health-related quality of life and patient mobility [5–7]. Furthermore, mortality risk at 10 years is twice as high in patients who had a foot ulcer compared to those who have not [8]. Besides the patient and social burden of diabetic foot disease, foot ulcers cost €5000 to €17,000 per episode in specialized centers in Europe and place a large burden on the health care systems [9].

Recognizing the potential for severe morbidity and high treatment costs related to foot ulceration, many experts call for widespread establishment of preventative foot care programs for persons with diabetes [10–12]. The most common mechanism of foot ulceration involves a cumulative effect of repetitive trauma at pressure points on the foot over the course of several days that goes unrecognized because of the presence of neuropathy [1]. Guidelines therefore recommend proper patient education, identification and treatment of the diabetic foot at-risk, integrated foot care and protective pressure-relieving footwear [10, 13, 14]*.* Despite these guidelines, the incidence of foot ulcer recurrence remains very high: 40% in the first year and 60% in the first 3 years after healing of a foot ulcer [1]. Therefore, care providers and patients are in need of new adjunctive ways to prevent ulcer recurrence.

Stimulated by the need for innovation in foot ulcer prevention, at the beginning of this millennium, researchers developed the concept of at-home monitoring of foot temperatures as a preventative tool [15–17]. Foot ulcers are preceded by increased local skin temperature due to inflammation and enzymatic autolysis of tissue as a result of being ambulatory [16, 18]; the foot tends to locally heat up before it breaks down. These increased temperatures can easily be assessed by the patients themselves using some form of thermometry that measures skin temperature at predefined regions of the foot [16]. By monitoring these temperatures on a frequent basis (preferably daily), the patient can identify signs of inflammation and impending ulceration. Timely identification of these warning signs allows the patient or care provider to take action to decrease the inflammation before an ulcer develops; for example, by reducing ambulatory activity, and/ or providing (further) offloading of the specific regions with footwear, orthoses or felted foam. In three randomized controlled trials (RCTs), such at-home monitoring of the foot temperature was shown to be a highly effective tool in preventing foot ulcer recurrence in patients with diabetes [15–17].

Despite the demonstrated efficacy of at-home monitoring of foot temperature in these studies [15–17], the intervention is currently not widely applied in (Dutch) health care. This may be because the external validity of the findings of these studies has not been proven to date, as the studies were conducted by the same research group in one geographical location in the United States (US). Recently, another RCT that followed a similar study protocol and used the same infrared temperature device as the previous US studies was conducted in Norway [19]. They found no statistical difference in ulcer recurrence rate between patients who monitored their skin temperature at home and patients who did not. However, only 41 patients were included in this study and the follow-up time was only 1 year, which means that this study was underpowered and caution is needed in interpreting these findings [19]. Another reason for the limited implementation in daily foot care may be that the intervention involves the purchase of a thermometer, while it is unclear whether the costs are reimbursed or have to be covered by the patient. And when “hot-spots” occur, additional diagnosis and treatment may be needed, of which frequency, costs and reimbursement are all unknown. Furthermore, the daily assessment and recording of foot temperatures may be seen as cumbersome and a heavy load in a situation where patients already have to monitor many aspects of their disease (e.g., glucose monitoring, insulin application, medicine intake, frequent check-ups, footwear use, etc.). Additionally, the knowledge on diagnostic accuracy of foot temperature assessments (e.g., false-positive and false-negative outcomes) is limited. The aforementioned US studies [15–17] did not report false-positive or false-negative outcomes. Eight of the 21 intervention-group patients in the Norwegian RCT measured an increased skin temperature one or more times during that study, but only four of these patients contacted the study nurse, and none developed a foot ulcer [19]. Recently, Frykberg and colleagues showed that with the use of a plantar foot-temperature-monitoring mat, 97% of all non-traumatic diabetic foot ulcers that developed in a group of 132 patients with a history of foot ulceration could be identified before development of the ulcer through a temperature difference > 2.2 °C between similar spots on both feet [20]. However, a high false-positive rate was also found (57% of temperature differences of > 2.2 °C found were false alarms), and the variation of contralateral temperature differences in the group of patients that did not develop a foot ulcer was substantial: 2.81 °C (±1.42 °C). Similar high rates of false-positive outcomes were also described by Wijlens and colleagues [21]. When such incorrect observations are made, they can result either in over-diagnosis or over-treatment resulting in an additional burden for both the patient and the health care system. For these reasons, it is important that more knowledge is gained in different settings on the effectiveness, cost-effectiveness, cost-utility, and diagnostic accuracy of using at-home temperature monitoring in high-risk patients with diabetes.

The International Working Group on the Diabetic Foot (IWGDF) identified ulcer prevention as an area where data on the effectiveness of interventions is scarce and data on their cost-effectiveness is lacking [10–12]. A better understanding of how (recurrent) foot ulcers develop and how they can be prevented in a cost-effective way, has major relevance for the patient and health care. Therefore, we have designed the Diabetic Foot Temperature Trial (DIATEMP). DIATEMP aims to assess the effectiveness, cost-effectiveness and cost-utility of at-home infrared foot-temperature monitoring to reduce the incidence of foot ulcer recurrence in patients with diabetes mellitus.

## Methods

### Primary objective

To evaluate the effectiveness, cost-effectiveness and cost-utility of daily at-home infrared plantar foot temperature monitoring to reduce the incidence of foot ulcer recurrence in patients with diabetes mellitus.

### Hypothesis

We hypothesize that enhanced therapy, which includes at-home infrared temperature monitoring of the foot, results in a significantly lower proportion of patients with foot ulcer recurrence, is cost-effective and saves costs per quality-adjusted life years (QALYs) gained when compared to usual care. The hypothesis is based on superiority of enhanced therapy compared to usual care.

### Standard protocol items

The DIATEMP trial protocol was written in accordance with the Standard Protocol Items: Recommendations for Interventional Trials (SPIRIT). The SPIRIT 2013 Checklist has been included as Additional file 1.

### Study design

The study design is a multicenter, outcome-assessor-blinded, parallel-group RCT with two study arms:

1. Enhanced therapy, including usual care as provided in the Netherlands and additional at-home daily plantar foot temperature monitoring
2. Usual care as provided in the Netherlands

**Fig. 1:**
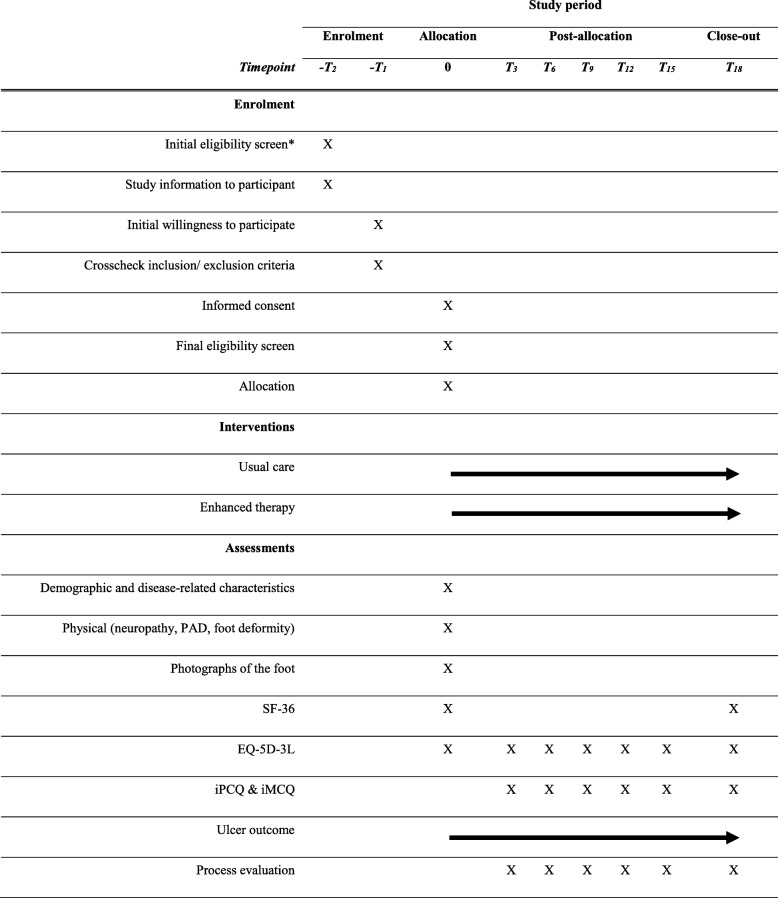
Standard Protocol Items: Recommendations for Interventional Trial (SPIRIT) Figure, study design overview. *Done by podiatrist during outpatient clinic visit, or retrospectively, from outpatient visit lists. *T*_*3,*_ *T*_*6,*_ *T*_*9*_,…refer to assessments at 3, 6, 9,…months’ follow-up. *PAD* peripheral arterial disease, *SF-36* 36-Item Short Form Health Survey, *EQ-5D-3 L*  3-Level EuroQol Quality of Life Scale, *iPCQ* Medical Technology Assessment (iMTA) Productivity Cost Questionnaire, *iMCQ* iMTA Medical Consumption Questionnaire

Patient recruitment takes place from seven university- or community-based hospitals with a multidisciplinary diabetic foot clinic in different regions throughout the Netherlands and from professional practices of podiatrists who participate in these multidisciplinary teams. Each diabetic foot clinic will operate as one of the study centers. Within each center, a physician and a podiatrist, both members of the diabetic foot team, will be involved. The participating hospitals are: Academic Medical Center (Amsterdam), VU Medical Center (Amsterdam), Ziekenhuisgroep Twente (Almelo), Maxima Medisch Centrum (Veldhoven), Maastricht University Medical Center (Maastricht), Reinier de Graaf Gasthuis (Delft), and Medisch Spectrum Twente (Enschede). Participants who consent to participate and who meet the inclusion and not the exclusion criteria will be randomized to the usual care or the enhanced therapy group. Each participant will be followed for 18 months or until a foot ulcer develops, after which the participant will be followed for the remainder of 18 months for the cost analysis only. The SPIRIT Figure (Fig. 1) shows an overview of the study design and the main procedures that participants will undergo during the course of the study.

### Participants

The study population consists of patients that are at high-risk of developing a foot ulcer. These are diabetic patients with a loss of protective sensation based on peripheral neuropathy and a history of foot ulceration in the 4 years prior to inclusion in the study, or a history of Charcot neuro-osteoarthropathy (IWGDF diabetic foot risk classification category 3 [22]).

### Inclusion and exclusion criteria

In order to be eligible to participate in this study, a participant must meet all of the following inclusion criteria:

1. Diagnosis of diabetes mellitus types 1 or 2
2. Aged 18 years or above
3. Have loss of protective sensation based in the presence of peripheral neuropathy [22]
4. Have a recent history of a foot ulcer or foot amputation, i.e., an ulcer, defined as cutaneous erosion through the dermis without reference to time present [22, 23], has been present for at least 2 weeks and has healed within 4 years before randomization; or a confirmed diagnosis of midfoot or forefoot Charcot neuro-osteoarthropathy
5. Ability to provide informed consent
6. Ambulatory status (i.e., not permanently wheel-chair bound)
7. The participant has foot care from a podiatrist or is willing to undergo foot care by a podiatrist

And not have any of the following exclusion criteria:

1. Active foot ulceration or open amputation sites
2. Active Charcot neuro-osteoarthropathy
3. Active foot infection, based on criteria of the Perfusion, Extent, Depth, Infection, and Sensation (PEDIS) classification [23]
4. Amputation proximal to the Chopart joint in both feet
5. Critical limb ischemia, based on criteria of the PEDIS classification [23]
6. Severe illness that would make 18 months’ survival unlikely, based on the clinical judgment by the physician
7. Concomitant severe physical or mental condition(s) that limit the ability to follow instructions for the study, based on the clinical judgment by the physician. This includes the inability to perform temperature measurements, without having a caretaker who can perform the temperature measurements
8. Current use of at-home foot temperature monitoring

### Sample size calculation

Based on the results of a footwear efficacy trial that was largely conducted in the same centers as this trial and also assessed patients with a history of foot ulceration [24], we anticipate for the usual-care group that 44% of participants will develop a recurrent foot ulcer during 18 months’ follow-up. Using a conservative estimate from three previous trials on the effectiveness of at-home foot temperature monitoring [15–17], we anticipate that 28.6% of participants in the enhanced therapy group will develop a recurrent foot ulcer in 18 months; this represents a 35% effect size. With α = 0.05 (two-sided), power 80%, and based on an intention-to-treat analysis in which clinical outcome data from all included patients will be assessed, 304 eligible participants are required and will be randomly assigned.

### Randomization and blinding

After the baseline assessment, participants will be randomly assigned to either usual care or enhanced therapy using an online-accessible, computer-generated allocation sequence (TENALEA Clinical Trial Data Management System; National Cancer Institute, Amsterdam, The Netherlands) that uses the nondeterministic minimization method. The allocation sequence will be prepared and managed by a noninvolved investigator from the Clinical Research Unit of the Academic Medical Center in Amsterdam. Randomization will be stratified according to participating center and gender.

The persons responsible for assessing the primary clinical outcome (i.e., foot ulcer) will be blinded to the group allocation. Participants are asked not to disclose their allocation in the study to their treating physician. The involved podiatrists and investigators are not blinded to group allocation.

### Usual care

Usual care as provided in the Netherlands generally follows universal guidelines [10, 13, 25], and consists of, but is not limited to:

1. Therapeutic (custom-made) footwear that is evaluated every 3 to 6 months by a medical specialist and/ or professional (e.g., orthotist, podiatrist). Footwear can include custom-made shoes, semi-custom-made shoes, and orthopedic appliances to footwear or podiatric insoles
2. Patient education that is provided by verbal and/ or written information by physician, podiatrist or the investigator during the baseline visit. Information addresses ulcer etiology, risk factors for ulceration, and self-care practices
3. Once every 3–6 months multidisciplinary foot care and screening and/ or once every 1–3 months preventative foot care and screening by a podiatrist and/ or diabetes pedicure

Participants are advised to contact their podiatrist if they identify an area of concern.

### Enhanced therapy

Enhanced therapy consists of:

- Usual care (see above), *and*
- At-home daily measurement of foot temperatures with an infrared thermometer on six predefined plantar regions on each foot, plus an additional one or two locations based on participants’ ulcer history or pre-ulcer status, if indicated and if different from the predefined locations

Using an infrared thermometer (TempTouch®, Diabetica Solutions, San Antonio, TX, USA) [15–17], skin temperature is measured at six predefined locations on the plantar surface of each foot: hallux, second and third toes, first, third, and fifth metatarsal heads. In addition, based on the participants’ ulcer history or pre-ulcer status, a maximum of two plantar foot regions can be added to the six predefined locations. For example, a midfoot region could be added for a person with a Charcot deformity.

The foot temperature will be measured once per day at both feet, per instruction in the morning directly after waking up. To standardize measurements, a video for the correct use of the thermometer has been developed and is shown to the participants in the enhanced therapy group during the baseline visit. To facilitate measurements and to facilitate adherence to measuring foot temperatures, the participant is advised to place the thermometer, logbook, and a pen on their bedside table. The participant will record each temperature value in a logbook. The participant will be asked to return completed logbooks to the coordinating study center (Academical Medical Center) every 4 weeks. Participants will receive once in 2 weeks a text reminder on their mobile phone to stimulate adherence in temperature monitoring and to remind them to decrease their ambulatory status if skin temperature in a region is > 2.2 °C compared to the corresponding region on the contralateral foot for two consecutive days.

If skin temperature measured in a region is > 2.2 °C compared to the corresponding region on the contralateral foot for two consecutive days, the participant is instructed to contact their podiatrist. The podiatrist will ask them about any swelling, change in color, change in structure, or drainage present at the high-temperature location. Based on these outcomes, further diagnosis at the podiatrist’s office may take place. In any case, the participant will be asked to decrease ambulatory activity with approximately 50% until the temperatures normalize (≤ 2.2 °C temperature difference) [15–17]. If the temperature difference exceeds 4 °C, or if temperatures do not normalize and are abnormal for four consecutive days, the participant is advised to arrange to be seen immediately by their podiatrist. If pre-signs of a foot ulcer are identified by the podiatrist, necessary precautions will be taken. This may include further offloading with therapeutic footwear or insoles, orthoses, felted foam or debridement. If needed, direct referral for treatment to specialized multidisciplinary care will take place. This may involve, among other things, immobilization of the foot.

During the first 2 weeks after randomization, patients are instructed to contact their local study investigator in case of abnormal (> 2.2 °C) temperature differences at the predefined regions on two or more consecutive days. These measurements may reveal structural temperature differences (> 2.2 °C) between the regions of interest of both feet without any symptoms or signs of inflammation or ulceration present (e.g., due to mild to moderate unilateral peripheral arterial disease). In these cases an individually calculated threshold temperature will be used based on the mean temperature difference between the left and right foot measured in the first 2 weeks after randomization.

Participants who are unable to measure skin temperature at the standard predefined regions due to amputation will measure at an alternative region to replace the amputated site according to a specifically designed amputation protocol (Table 1).

**Table 1:** Amputation protocol

Amputation site	Alternative region for measurement on the ipsilateral foot	Region(s) for comparison on the contralateral foot
Hallux	MTH 1 or 2nd toe^a^	Hallux
2nd toe	3rd toe	2nd toe
3rd toe	2nd or 4th toe^a^	3rd toe
Hallux and (trans)metatarsal I	- Most distal plantar part of the amputation site or 2nd toe^a^	- Hallux
	- Most distal plantar part of the amputation site	- MTH 1
2nd toe and (trans)metatarsal II	3rd toe	2nd toe
3rd toe and (trans)metatarsal III	- 4th toe	- 3rd toe
	- Most distal plantar part of the amputation site	- MTH 3
5th toe and (trans)metatarsal V	Most distal plantar part of the amputation site	MTH 5
Transmetatarsal amputation of the forefoot	Most distal plantar part of the amputation site at the base of the 1st, 3rd, and 5th metatarsals	MTH 1, 3, and 5^b^
Amputation of the forefoot through the Lisfranc joint	The plantar site of the 1st cuneiform bone, 3rd cuneiform bone, and the cuboid bone	MTH 1, 3, and 5^b^
Amputation of the forefoot through the Chopart joint	Most distal plantar part of the amputation site: medial, mid, and lateral	MTH 1, 3, and 5^b^

^a^Based on temperature values in the first 2 weeks, the investigator chooses the alternative region

^b^In case of a transmetatarsal amputation of the forefoot, or a more proximal amputation, no alternative region to measure for the hallux, 2nd and 3rd toes can be identified. In these cases, the measured temperatures of these regions in the intact foot are compared with the mean temperature of these regions measured in the first 2 weeks of temperature monitoring, using the same foot as reference. This is comparable to the protocol used for participants with a unilateral transtibial amputation. For further explanation, see the text

If participants have a transtibial or more proximal amputation, plantar foot temperatures at the predefinedregions of the intact foot will be compared to a calculated mean temperature of the same regions as obtained during the first 2 weeks of measurement after randomization. The investigator calculates the mean temperature for each region over the first 2 weeks, enters these as reference in the logbooks of the participant and sends the logbooks to the participant. Starting in the third week, participants compare their daily temperatures with these new reference temperatures. The same threshold temperature (> 2.2 °C) applies.

### Outcomes

The primary outcomes in this study are the cost (savings) per patient without a foot ulcer (i.e., cost-effectiveness) and per QALY gained (i.e., cost-utility). The primary clinical outcome is the proportion of participants with a recurrent foot ulcer on the plantar foot, apical surfaces of the toes, interdigital spaces or medial and lateral forefoot surfaces during 18-month follow-up. A foot ulcer is defined as a cutaneous erosion through the dermis without reference to time present [22, 23]. Endpoints in the study are either a foot ulcer, or 18 months of follow-up.

Secondary outcomes are the costs of therapy and of ulcer treatment, adherence to at-home foot temperature monitoring, and a multivariate risk score for ulcer recurrence.

### Study procedures

The study investigators will obtain informed consent and will perform all study measurements, during baseline and the 3-month semi-structured interviews with participants by phone.

#### Baseline assessment

After providing informed consent, participants will undergo a baseline assessment at their study center to confirm definitive eligibility for inclusion in the study. The following characteristics will be obtained during the baseline visit:

1. Demographic information and disease-related characteristics (e.g., diabetes duration and control, presence of complications, ulcer history, footwear use, etc.);
2. Peripheral neuropathy assessment:
  - Presence of neuropathy will be assessed by measuring the loss of protective sensation by using the 10-g (5.07) Semmes-Weinstein monofilament at the plantar surface of the hallux and the first and fifth metatarsal heads of both feet [10]. Neuropathy is defined when the monofilament is not felt on two or more locations [22]
  - A 128-Hz tuning fork held on the apex of the great toe [10]. Neuropathy is defined when the participant indicates not feeling the vibration [22]
3. Peripheral vascular assessment by palpation of the dorsalis pedis and posterior tibial pulses of both feet, according to the PEDIS classification system [23]. If pulses are not palpable, additional assessment of peripheral vascular status will be done by measuring toe pressures or the participant’s medical record is checked for their vascular status
4. Presence of foot deformity will be assessed clinically. These include hammer/ claw toes, prominent metatarsal heads, hallux valgus, pes planus, pes cavus, Charcot deformity, and any amputation. A participant’s feet will be classified into one of four categories according to the severity of deformity present: no deformity, mild deformity, moderate deformity, and severe deformity [24]

If definitive eligibility has been confirmed, photographs of the plantar and dorsal surfaces of both feet will be taken according to a standardized protocol [24], and health-related quality of life will be assessed by using the 36-Item Short Form Health Survey (SF-36) and the 3-Level EuroQol Quality of Life Scale (EQ-5D-3 L) questionnaires.

#### Ulceration

If the participant, treating physician, podiatrist or pedicure identifies an ulcer between regular study visits, they are instructed to inform the diabetic foot team or podiatrist immediately, and have photographs taken of the foot. The podiatrist will take photographs of the wound, debride the wound if required to assess outcome, classify the ulcer using the University of Texas system and the PEDIS classification system, and again take photographs of the lesion after debridement using a standardized protocol and enter all data in an outcome Case Report Form (CRF) [23, 26]. This information will be sent to the investigator, who will upload all information anonymously to a web-based environment for ulcer outcome assessment by a panel of, minimally, three blinded and independently operating foot care specialists that will determine the definitive outcome [24].

#### Health-related quality of life and costs

For the cost-effectiveness and cost-utility analysis, the following data will be collected at 3-monthly intervals:

1. Health-related quality of life will be assessed by asking participants to complete the EQ-5D-3 L questionnaire. These questionnaires will be sent to the participant’s home and returned after completion in an enclosed return-envelope
2. At the same time-interval of 3 months, or in case a foot ulcer develops at monthly intervals, the participant is asked to complete the study-specified versions of the Institute for Medical Technology Assessment (iMTA) Productivity Cost Questionnaire (iPCQ) and the iMTA Medical Consumption Questionnaire (iMCQ) [27] to gather volume data on productivity loss, out-of-hospital use of health care resources (e.g., podiatrist, pedicure), and out-of-pocket expenses
3. Use of intramural health care resources during the study will be obtained from the participants’ medical status

#### Process evaluation

At 3-monthly intervals, the investigator will contact the participant by phone to conduct a process evaluation of the intervention. Intervention group participants will be asked in a semi-structured interview about their experiences with at-home temperature monitoring. All participants will also be asked about contacts with health care professionals and any foot problems encountered in the previous 3-month period to crosscheck for the completed iPCQ and iMCQ questionnaires and about any lesion that may have developed.

### Data management

The participants will be coded by the number of the participating center (two digits) followed by the number of the participant (three digits). All information referring to the patients will be saved in a locked record office or on a computer with password security. Only the investigators have access to this study information. Name and date of birth of the participants will only be recorded on the informed consent form, which will be kept in a locked cupboard with the lead investigator per center, separate from the digital data and without a possibility to trace the data. All study data will be entered anonymized in an electronic database OpenClinica®. All study information will be saved for at least 15 years after the study has ended.

### Monitoring

Given the pragmatic nature of the intervention and the very low, negligible, risk for the participants in the study, an independent Data Safety and Monitoring Board has not been established. The investigators are responsible for procedures of data monitoring. To facilitate compliance with Good Clinical Practice guidelines, the investigator will permit study-related monitoring, audits, and inspections by authorized organizations. Aspects that will be monitored may include: inclusion rate; trial master file; informed consent progress; inclusion and exclusion criteria; source data verification; safety reporting; investigational product; trial procedures; and closing and reporting. Currently, the DIATEMP trial is monitored internally by the Academic Medical Center Amsterdam, VU Medical Center and the Maastricht University Medical Center. The role of the data monitor is to review study documentation, CRFs, and informed consents.

### Withdrawal of participants

Participants can withdraw from participation in the study at any time for any reason if they wish to do so, and without any consequences for their normal care. The physician can decide to withdraw a participant from the study in case of urgent medical reasons. After withdrawal from the study, information on ulcer outcome at 18 months will be obtained from the participant’s medical record if the participant consents to this procedure. Ulcer outcome data from participants who die during the study will be based on outcome at the moment of death (last observation carried forward).

### Serious adverse events (SAEs)

Any SAE that occurs during the study will be reported by the principal investigator to the accredited Medical Research Ethics Committee (METC) that approved the protocol within 15 days of when the principal investigator has been informed about the serious adverse event (within 7 days if death is the SAE).

### Statistical analysis

Statistical analysis will be performed after the last follow-up visit of the last participant in the study, and will be conducted using SPSS statistical software (IBM Corporation, Armonk, NY, USA). All tests will assess group effects, will be two-sided, and use *P* < 0.05 as significance level. All comparisons between groups are based on both an intention-to-treat and a per-protocol analysis.

Effectiveness of the intervention will be assessed using chi-square analysis. A competing risk analysis will be done to assess the difference by time to ulcer recurrence, with unrelated death as the competing risk and absence of ulcer at 18 months as censored observation.

### Economic evaluation

The economic evaluation will be performed as a cost-effectiveness analysis with the costs per prevented foot ulcer as the primary outcome. A cost-utility analysis will be performed with the costs per QALY as outcome. Both will be performed from a societal perspective. Considering the time horizon of 18 months, we will discount the effects and costs during the second year of follow-up. The Dutch Government recommends a discount rate of 4% for costs and 1.5% for effects [28].

Given the societal perspective, data will be collected on direct medical and non-medical costs as well as indirect non-medical costs. Direct medical costs include, for example, the costs of foot care, the thermometer and care provided by other health care professionals (general practitioner, medical specialist). Direct non-medical costs include, for example, out-of-pocket expenses by patients for travel to and from health care providers, private household assistance, and over-the-counter medication. Indirect non-medical costs reflect the costs of productivity loss due to sick leave from work or lower productivity while at work. Costs will be calculated as the product sum of resource volume data and their respective unit costs, as described in the Dutch manual for costing in health care research [29]. Costs associated with productivity loss will be based on the friction cost method, applying the actual mean friction period in the base year of the study. After price-indexing with general yearly consumer price indices, all costs will be expressed in euros for the base year 2015.

Incremental cost-effectiveness ratios will be calculated as the extra costs per additional patient without foot ulcer and the extra costs per QALY gained. To account for sampling variability, group differences will be assessed by calculating the 95% confidence intervals after correction for bias and using accelerated non-parametric bootstrapping. If enhanced therapy does not dominate usual care, results will be displayed graphically with cost-effectiveness acceptability curves for willingness to pay values up to €100,000.

Health utilities associated with the scoring profiles on the EQ-5D-3 L are available through the cross-walk value sets from the www.euroqol.org website and will be used to derive a QALY estimate for each patient. This QALY will be calculated as the product sum of health utilities and the lengths of the periods in-between successive measurements. In case of missing assessments, the last observation will be carried forward. Sensitivity analyses will be performed for different (Dutch and UK population-based) health-utility scoring algorithms used to derive QALYs as well as for different discounting rates to reflect time preference.

A subgroup analysis of cost-effectiveness and cost-utility will be performed by level of adherence to temperature monitoring.

The cost consequences of monitoring foot temperature at home, such as by the use of the measurement device and intensified monitoring costs, may affect health care budgets. A budget impact analysis (BIA) will be carried out from governmental, health care provider, and insurer perspectives. The governmental perspective is chosen to help setting priorities in health care optimization while simultaneously considering the wider implications of stimulating enhanced therapy for diabetic patients at a high risk of ulcers beyond the health care sector. The provider perspective is chosen to support local decisions on economies of scale and affordability. The insurer perspective is chosen to assess the net financial consequences of offering intensified monitoring to high-risk patients who have a history of ulceration, which may help to shift health care use from the second to the first echelon. For this study, the BIA will be conducted using a decision-tree model developed in Microsoft® Excel. The BIA will be performed according to the ISPOR Task Force principles [30].

Finally, a scenario analysis will be carried out, simulating three implementation scenarios against the base scenario (usual care): (1) immediate use of the device, (2) gradual use (an absolute 25% yearly increase of patients in the target group using the device), and (3) partial use (up to 70% of the whole target population). Sensitivity analyses will be applied for the level of adherence to temperature monitoring and for a potential shift from podiatric to pedicure foot care. The BIA will have a time horizon of 4 years. Results will be reported for successive calendar years.

## Discussion

The DIATEMP trial is a multicenter RCT with the aim to determine cost-effectiveness and cost-utility of at-home monitoring of plantar foot temperature for preventing foot ulcer recurrence in high-risk diabetes patients. Following three successful RCTs demonstrating the efficacy of at-home foot temperature for preventing diabetic foot ulcer recurrence in one geographical region (Texas) in the US [15–17], this is the first adequately designed and powered RCT to investigate this intervention in another geographical location (the Netherlands). In addition to the previous RCTs, we include assessment of cost-effectiveness and cost-utility. After the start of participant inclusion in the study, we modified and improved our protocol to a limited extent based on new insights and necessities; the most important changes are described and clarified below.

Crucial in any trial is sufficient patient recruitment. We anticipated, based on calculations of recruitment rate from a previous trial [24], that the required period for including the 304 participants would take 15 months in the participating five centers. Unfortunately, the response rate of potentially eligible participants was below 25%, while we hypothesized a response rate of approximately 50%. To increase participant inclusion, we intensified the collaboration with the study centers and the involved podiatrists, and we added two more study centers (VU Medical Center, Amsterdam, and Medisch Spectrum Twente, Enschede). We additionally adjusted one of the inclusion criteria. We initially included only participants with a healed foot ulcer in the 2 years prior to study randomization. This had the advantage of selecting only the highest-risk patients, with re-ulceration rates being approximately 60% in the first 3 years after healing [1]. To increase the potential for inclusion we prolonged the ulcer-free period before study randomization to a maximum of 4 years. These changes in the protocol resulted in increased recruitment rates for the trial.

Due to the high risk of ulceration and frequent occurrence, diabetic patients with a history of amputation are important to include in a prevention trial [2]. In the trial of Lavery and colleagues, patients with a minor amputation, such as a great toe, were instructed to measure their foot temperature at the basis of the amputated region, while patients with an amputation proximal of the forefoot were excluded [17]. Other trials on at-home monitoring of skin temperature describe no specific protocol for patients with an amputation [15, 16, 19]. Since at-home foot temperature monitoring is based on the principle of comparing bilateral foot temperatures at the same anatomical region, a specific protocol is needed for participants with an amputation. Initially, we used the protocol of Lavery and colleagues as described above; however, this often resulted in participants finding temperature differences that were consistently above 1.5 °C in the first 2 weeks of monitoring, increasing the potential for false-positive outcomes. These high temperature differences occur due to the changed anatomy and biomechanics following amputation, with tissue stress and temperature being structurally higher at the stump location. We modified our measurement protocol to take such systematic differences into account, as described in the methodology. Consequently, only participants with a bilateral amputation proximal to the Chopart joint had to be excluded from participation in the trial.

The measurement protocol in our trial was largely based on previous trials of Lavery and Armstrong and colleagues, in which six predefined regions of interest were measured: hallux, first, third, and fifth metatarsal heads, midfoot, and hindfoot [15–17]. Since many foot ulcers occur at the toes, and re-ulceration occurs mostly at the previous ulcer location [31], we added the option of measuring a maximum of two regions of interest in addition to the standard six, to provide a solution for previous foot ulcers or signs of pre-ulceration (e.g., abundant callus, subcutaneous hemorrhage or blister) being present at toes two to five. During the trial we noticed (blinded to group allocation) that ulcers did not develop at the midfoot or hindfoot. Therefore, in October 2017, we modified the six standard regions of interest to include the plantar surface of the second and third toes instead of the midfoot and hindfoot [20, 24]. For participants with a high risk of developing a foot ulcer at the midfoot or hindfoot, such as in midfoot Charcot deformity, this region would still be selected for temperature measurement as an additional region of interest.

A strength of this trial is that, in addition to assessing effectiveness in preventing foot ulcer recurrence, we assess cost-effectiveness and cost-utility of the procedure. These outcomes are important given the extra investment in measurement equipment and time of the health care professional and the patient in monitoring the foot. Another strength is that not just any foot ulcer, but only plantar foot ulcers and ulcers that develop at the apex of the toes, the interdigital spaces, and the lateral and medial forefoot are the primary clinical outcome. These locations are often subject to foot ulceration as a result of repetitive mechanical stress due to deformity present and rubbing of the toes. If inflammation occurs at these areas before foot ulceration develops, we anticipate that the temperature increase due to the inflammation is being measured at one of the measurement locations on the foot.

In conclusion, the DIATEMP trial aims to provide level-1 evidence for the effectiveness, cost-effectiveness and cost-utility of at-home monitoring of foot skin temperature to prevent foot ulcer recurrence in high-risk diabetes patients. The outcomes of this RCT, together with analyses on the usability and implementability of the intervention, is expected to have impact on the use of foot temperature monitoring and the design of foot temperature monitoring systems as method for self-management to prevent diabetic foot complications in high-risk patients with diabetes.

## Trial status

Netherlands Trial Registry, ID: NTR5403. Registered on 8 September 2015. The trial commenced recruitment in November 2015 and recruitment is expected to be completed in July 2018.

## Additional file

Additional file 1: Standard Protocol Items: Recommendations for Interventional Trial (SPIRIT) 2013 Checklist: recommended items to address in a clinical trial protocol and related documents. (PDF 57 kb)

## Abbreviations

BIA: Budget impact analysis
CRF: Case Report Form
DIATEMP: Diabetic Foot Temperature Trial
EQ-5D-3 L: 3-Level EuroQol Quality of Life Scale
iMCQ: Medical Consumption Questionnaire
iMTA: Institute for Medical Technology Assessment
iPCQ: Productivity Cost Questionnaire
ISPOR: International Society for Pharmacoeconomics and Outcomes Research
IWGDF: International Working Group on the Diabetic Foot
METC: Medical Research Ethics Committee; in Dutch: Medisch Ethische Toetsings Commissie
PAD: Peripheral arterial disease
PEDIS classification: Perfusion, Extent, Depth, Infection, and Sensation classification
QALY: Quality-adjusted life year
RCT: Randomized controlled trial
SAE: Serious adverse events
SF-36: 36-Item Short Form Health Survey
SPIRIT: Standard Protocol Items: Recommendations for Interventional Trials

## References

[1] Armstrong DG, Boulton AJM, Bus SA (2017). Diabetic foot ulcers and their recurrence. N Engl J Med.

[2] Pecoraro RE, Reiber GE, Burgess EM (1990). Pathways to diabetic limb amputation. Basic for prevention. Diabetes Care.

[3] Abbott CA, Carrington AL, Ashe H, Bath S, Ever LC, Griffiths J (2002). The North-West Diabetes Foot Care Study: incidence of, and risk factors for, new diabetic foot ulceration in a community-based patient cohort. Diabet Med.

[4] Muller IS, de Grauw WJ, van Gerwen WH, Bartelink ML, van den Hoogen HJ, Rutten GE (2002). Foot ulceration and lower limb amputation in type 2 diabetic patients in Dutch primary health care. Diabetes Care.

[5] Boulton AJ, Kirsner RS, Vileikyte L (2004). Clinical practice. Neuropathic diabetic foot ulcers. N Engl J Med.

[6] Gonzalez JS, Vileikyte L, Ulbrecht JS, Rubin RR, Garrow AP, Delgado C (2010). Depression predicts first but not recurrent diabetic foot ulcers. Diabetologia.

[7] Kerr M, Rayman G, Jeffcoate WJ (2014). Cost of diabetic foot disease to the National Health Service in England. Diabet Med.

[8] Iversen MM, Tell GS, Riise T, Hanestad BR, Østbye T, Graue M (2009). History of foot ulcer increases mortality among individuals with diabetes: ten-year follow-up of the Nord-Trøndelag health study, Norway. Diabetes Care.

[9] Prompers L, Huijberts M, Schaper N, Apelqvist J, Bakker K, Edmonds M (2008). Resource utilisation and costs associated with the treatment of diabetic foot ulcers. Prospective data from the Eurodiale study. Diabetologia.

[10] Bus SA, Van Netten JJ, Lavery LA, Monteiro-Soares M, Rasmussen A, Jubiz Y (2016). IWGDF guidance on the prevention of foot ulcers in at-risk patients with diabetes. Diabetes Metab Res Rev.

[11] Bus SA, Van Netten JJ (2016). A shift in priority in diabetic foot care and research: 75% of foot ulcers are preventable. Diabetes Metab Res Rev.

[12] Jeffcoate WJ, Vileikyte L, Boyko EJ, Armstrong DG, Boulton AJM (2018). Current challenges and opportunities in the prevention and management of diabetic foot ulcers. Diabetes Care.

[13] Bakker K, Apelqvist J, Lipsky BA, Van Netten JJ (2016). Foot IWGotD. The 2015 IWGDF guidance documents on prevention and management of foot problems in diabetes: development of an evidence-based global consensus. Diabetes Metab Res Rev.

[14] Van Netten JJ, Price PE, Lavery L, Monteiro-Soares M, Rasmussen A, Jubiz Y (2016). Prevention of foot ulcers in the at-risk patient with diabetes: a systematic review. Diabetes Metab Res Rev.

[15] Armstrong DG, Holtz-Neiderer K, Wendel C, Mohler MJ, Kimbriel HR, Lavery LA (2007). Skin temperature monitoring reduces the risk for diabetic foot ulceration in high-risk patients. Am J Med.

[16] Lavery LA, Higgins KR, Lanctot DR, Constantinides GP, Zamorano RG, Athanasiou KA (2004). Home monitoring of foot skin temperatures to prevent ulceration. Diabetes Care.

[17] Lavery LA, Higgins KR, Lanctot DR, Constantinides GP, Zamorano RG, Athanasiou KA (2007). Preventing diabetic foot ulcer recurrence in high-risk patients: use of temperature monitoring as a self-assessment tool. Diabetes Care.

[18] Armstrong DG, Lavery LA, Liswood PJ, Todd WF, Tredwell JA (1997). Infrared dermal thermometry for the high-risk diabetic foot. Phys Ther.

[19] Skafjeld A, Iversen MM, Holme I, Ribu L, Hvaal K, Kilhovd BK (2015). A pilot study testing the feasibility of skin temperature monitoring to reduce recurrent foot ulcers in patients with diabetes—a randomized controlled trial. BMC Endocr Disord.

[20] Frykberg RG, Gordon IL, Reyzelman AM, Cazzell SM, Fitzgerald RH, Rothenberg GM (2017). Feasibility and efficacy of a smart mat technology to predict development of diabetic plantar ulcers. Diabetes Care.

[21] Wijlens AM, Holloway S, Bus SA, Van Netten JJ (2017). An explorative study on the validity of various definitions of a 2·2°C temperature threshold as warning signal for impending diabetic foot ulceration. Int Wound J.

[22] Schaper NC, Van Netten JJ, Apelqvist J, Lipsky BA, Bakker K (2016). Foot IWGotD. Prevention and management of foot problems in diabetes: a summary guidance for daily practice 2015, based on the IWGDF guidance documents. Diabetes Metab Res Rev.

[23] Schaper NC (2004). Diabetic foot ulcer classification system for research purposes: a progress report on criteria for including patients in research studies. Diabetes Metab Res Rev.

[24] Bus SA, Waaijman R, Arts M, de Haart M, Busch-Westbroek T, van Baal J (2013). Effect of custom-made footwear on foot ulcer recurrence in diabetes: a multicenter randomized controlled trial. Diabetes Care.

[25] Nederlandse Internisten Vereniging. Nederlandse Richtlijn Diabetische Voet, 2017 (Dutch). https://richtlijnendatabase.nl/richtlijn/diabetische_voet/.

[26] Armstrong DG, Lavery LA, Harkless LB (1998). Validation of a diabetic wound classification system. The contribution of depth, infection, and ischemia to risk of amputation. Diabetes Care.

[27] Bouwmans C, Hakkaart-van Roijen L, Koopmanschap M, Krol M, Severens H, Brouwer W, Rotterdam EU (2013). Manuals: Medical Consumption Questionnaire.

[28] Nederland Z. Richtlijn voor het uitvoeren van economische evaluaties in de gezondheidszorg, 2016 (Dutch). https://www.zorginstituutnederland.nl/publicaties/publicatie/2016/02/29/richtlijn-voor-het-uitvoeren-van-economische-evaluaties-in-de-gezondheidszorg.

[29] Hakkaart-van Roijen L, Van der Linden N, Bouwmans C, Kanters T, Tan SS. Kostenhandleiding: Methodologie van kostenonderzoek en referentieprijzen voor economische evaluaties in de gezondheidszorg: Institute for Medical Technology Assessment; 2016.

[30] Mauskopf JA, Sullivan SD, Annemans L, Caro J, Mullins CD, Nuijten M (2007). Principles of good practice for budget impact analysis: report of the ISPOR task force on good research practices—budget impact analysis. Value Health.

[31] Waaijman R, De Haart M, Arts MLJ, Wever D, Verlouw AJWE, Nollet F (2014). Risk factors for plantar foot ulcer recurrence in neuropathic diabetes patients. Diabetes Care.

